# miR-190 suppresses breast cancer metastasis by regulation of TGF-β-induced epithelial–mesenchymal transition

**DOI:** 10.1186/s12943-018-0818-9

**Published:** 2018-03-06

**Authors:** Yue Yu, Wei Luo, Zheng-Jun Yang, Jiang-Rui Chi, Yun-Rui Li, Yu Ding, Jie Ge, Xin Wang, Xu-Chen Cao

**Affiliations:** 1 The First Department of Breast Cancer, Tianjin Medical University Cancer Institute and Hospital, National Clinical Research Center for Cancer, Huanhuxi Road, Hexi District, Tianjin, 300060 China; 2 Key Laboratory of Cancer Prevention and Therapy, Tianjin, 300060 China; 3 Tianjin’s Clinical Research Center for Cancer, Tianjin, 300060 China; 4 Key Laboratory of Breast Cancer Prevention and Therapy, Tianjin Medical University, Ministry of Education, Tianjin, 300060 China

**Keywords:** Breast cancer, Transforming growth factor-β, Epithelial to mesenchymal transition, miR-190, SMAD2, ZEB1

## Abstract

**Background:**

Breast cancer is the most common cancer among women worldwide and metastasis is the leading cause of death among patients with breast cancer. The transforming growth factor-β (TGF-β) pathway plays critical roles during breast cancer epithelial–mesenchymal transition (EMT) and metastasis. SMAD2, a positive regulator of TGF-β signaling, promotes breast cancer metastasis through induction of EMT.

**Methods:**

The expression of miR-190 and SMAD2 in breast cancer tissues, adjacent normal breast tissues and cell lines were determined by RT-qPCR. The protein expression levels and localization were analyzed by western blotting and immunofluorescence. ChIP and dual-luciferase report assays were used to validate the regulation of ZEB1-miR-190-SMAD2 axis. The effect of miR-190 on breast cancer progression was investigated both in vitro and in vivo.

**Results:**

miR-190 down-regulation is required for TGF-β-induced EMT. miR-190 suppresses breast cancer metastasis both in vitro and in vivo by targeting SMAD2. miR-190 expression is down-regulated and inversely correlates with SMAD2 in breast cancer samples, and its expression level was associated with outcome in patients with breast cancer. Furthermore, miR-190 is transcriptionally regulated by ZEB1.

**Conclusions:**

Our data uncover the ZEB1-miR-190-SMAD2 axis and provide a mechanism to explain the TGF-β network in breast cancer metastasis.

**Electronic supplementary material:**

The online version of this article (10.1186/s12943-018-0818-9) contains supplementary material, which is available to authorized users.

## Background

Breast cancer is the most common type of cancer among women worldwide. Approximately 252, 710 women are diagnosed with breast cancer annually, accounting for 30% of all cancers among women [1]. Although the rates of metastasis and mortality in patients with breast cancer have decreased, metastases at distant sites are still responsible for majority of the cancer deaths [2]. Distant disease involves multiple complex mechanisms, including invasion and migration, angiogenesis, anoikis resistance, and epithelial–mesenchymal transition (EMT) [3–5]. Therefore, a better understanding of such molecular mechanisms is required to facilitate the development of more accurate prognostic markers as well as effective therapeutic strategies [6]. Recently, the roles of microRNAs (miRNAs) have begun to be increasingly appreciated among the many molecular players described to date in breast cancer metastasis.

The transforming growth factor-β (TGF-β) signaling pathway is a critical player in embryonic development and cellular homoeostasis in most species ranging from flies to mammals [7]. The TGF-β signaling cascade is initiated by binding of the ligands to type II receptors, which recruit and phosphorylate type I receptors. The activated type I receptors phosphorylate the intracellular effectors, SMAD2/SMAD3, which form complexes with SMAD4 and then shuttle into the nucleus for transcriptional regulation [8, 9]. The TGF-β signaling pathway plays critical roles in multiple cancer biological processes, including growth, migration, invasion, differentiation, apoptosis, stemness, angiogenesis, and modification of the microenvironment [10, 11]. The TGF-β-SMAD pathway induces breast cancer progression by regulation of multiple stages in the metastatic process, among which EMT is a well-studied process that endows tumor cells with increased aggressiveness. EMT is a developmental process whereby epithelial cells reprogram to a mesenchymal-like phenotype. It is driven by a set of transcription factors, including the basic helix-loop-helix factor, TWIST1/2, and the zinc finger factors, SNAI1/2 and ZEB1/2, which function as direct or indirect repressors of the epithelial marker E-cadherin (CDH1) and inducers of mesenchymal markers, such as vimentin, N-cadherin (CDH2), and fibronectin. The TGF-β signaling pathway regulates these transcription factors, which confers TGF-β a potent inducer of EMT [12, 13].

miRNAs are a class of small non-coding RNAs, which are believed to negatively regulate gene expression by binding to complementary sequences in the 3′ untranslated regions (UTRs) by translational inhibition and destabilization of target mRNAs [14, 15]. Increasing evidence supports that miRNAs are frequently dysregulated in breast cancer, and act as either oncogenes or tumor suppressors and critical regulators of carcinogenesis and cancer progression, as well as useful diagnostic and prognostic markers in breast cancer [6, 16, 17]. However, our understanding of how miRNAs regulate breast cancer development and progression, particularly how they affect breast cancer metastasis is still limited. miR-190 is located at an intron region of the TLN2 gene on chromosome 15q22.2. Previous reports have shown that its expression decreased in aggressive neuroblastomas and its overexpression leads to inhibition of tumor growth and prolonged dormancy periods in fast-growing tumors [18–20]. miR-190 attenuates the migration and invasion abilities of hepatocellular carcinoma cells through inhibition of EMT phenotype and inhibits tumor angiogenesis [21]. miR-190 is also involved in estrogen receptor signaling, causing inhibition of breast cancer metastasis [22]. These results suggest that miR-190 may act as a tumor suppressor. In contrast, miR-190 is up-regulated in gastric cancer tissues and contributes to gastric cancer progression [23]. This suggests that miR-190 may play a different role in different tumor environments and different stages of tumor development. More studies should be carried out on different tumor types.

Here, we investigate the role of miR-190 in breast cancer development and progression. miR-190 antagonizes TGF-β-induced EMT by targeting SMAD2 and suppresses breast cancer metastasis. miR-190 is transcriptionally regulated by an EMT-related transcription factor and forms a feedback loop with TGF-β/SMAD2 signaling. Therefore, our study reveals a novel mechanism of TGF-β signaling pathway during metastasis in breast cancer.

## Methods

### Cell culture

MCF10A, MCF7, T47D, BT474, MDA-MB-468, and MDA-MB-231 cell lines were obtained from the Cell Bank of the Chinese Academy of Sciences (Shanghai, China). MCF10A was cultured as previously described [24] in Dulbecco’s modified Eagle’s medium (DMEM)/F12 supplemented with 5% horse serum (Life Technologies, Grand Island, NY, USA), 10 μg/mL insulin, 0.5 μg/mL hydrocortisone, 20 ng/mL epidermal growth factor (EGF), 100 ng/mL cholera toxin, and penicillin/streptomycin. BT474 cells were maintained in RPMI-1640 medium supplemented with 15% fetal bovine serum (FBS) and 0.1 IU/mL insulin. MDA-MB-231 cells were cultured in Leibovitz’s L-15 medium supplemented with 10% FBS without CO_2_ at 37°C. T47D and MCF7 cells were cultured in DMEM supplemented with 10% FBS.

### Clinical samples

Breast cancer specimens were obtained from Tianjin Medical University Cancer Institute and Hospital (TMUCIH). A total of 200 paraffin-embedded specimens and 30 primary breast cancer tissue and paired adjacent normal breast tissue specimens were included in this study. All tumor samples were from patients with a newly diagnosed breast cancer who had received no therapy before sample collection. This study was approved by the Institutional Review Board of the Tianjin Medical University Cancer Institute and Hospital and written consent was obtained from all participants.

### Antibodies and reagents

The antibodies and reagents are listed in Additional file 1: Supplementary Materials and Methods.

### Plasmids, miRNA, and small interfering RNA (siRNA)

The plasmids, miRNA, and siRNA are described in Additional file 1: Supplementary Materials and Methods.

### Transient and stable transfection of breast cancer cells

For transient transfection, miRNA or siRNAs were transfected into different cell lines using FuGENE HD Transfection Reagent (Promega, Madison, WI, USA) and plasmids were transfected using TransFast Transfection Reagent (Promega) according to the manufacturer’s recommendations. To generate stable cells, the lentiviruses (RiboBio, Shanghai, China) were used to infect MDA-MB-231-luc cells according the manufacturer’s recommendations.

### Proliferation and invasion assays

Both MTT and plate colony formation assays were used to evaluate cell proliferation ability. Transwell assay was used to evaluate cell invasion. Experiments were carried our as described in Additional file 1: Supplementary Materials and Methods.

### Western blotting and immunofluorescence

Standard procedures for western blotting and immunofluorescence are described in Additional file 1: Supplementary Materials and Methods.

### RNA extraction and reverse transcription quantitative polymerase chain reaction (RT-qPCR)

Total RNA of cultured cells, surgically resected fresh breast tissues, and formalin-fixed paraffin-embedded clinical specimens were extracted using mirVana PARIS kit (Life Technologies) according to the manufacturer’s recommendations. qPCR was performed to detect mRNA expression using GoTaq qPCR Master Mix (Promega). TaqMan RT-qPCR was performed to detect mature miRNA expression using TaqMan miRNA reverse transcription kit, has- RNU6B (U6, ABI Assay ID: 001093) and miR-190 (ABI Assay ID: 000489) according to the manufacturer’s protocol (Life Technologies). The Ct values of each gene in triplicate reactions were averaged. Quantification of the target gene expression was calculated by normalizing the averaged Ct value of target gene to the averaged Ct value of housekeeping gene ACTB (ΔCt), and determined as 2-ΔCt. The sequences of PCR primers are listed in Additional file 1: Table S1.

### Chromatin immunoprecipitation (ChIP) analysis

ChIP assay was performed according to the protocol of Upstate Biotechnology as previously described [25]. The primer sequence used for miR-190 promoter was: 5′-GGTCTTTGATGATGATTCTGG-3′ and 5′-CTAGGCACAGTATTGAAGGTT-3′. Monoclonal antibody against HA was used for immunoprecipitation and normal IgG was used as negative control. Five percent of original DNA was used as input control.

### Luciferase reporter assays

Luciferase assays were carried out using a dual luciferase assay kit according to the manufacturer’s recommendations as previously described [25].

### Xenograft

The stable miR-190-overexpressed MDA-MB-231 and control cells (3 × 10^6^ cells) together with 100 μg of Matrigel (BD Biosciences, San Diego, CA, USA) were inoculated into the mammary fat pads of 5-week-old female SCID mice. Tumor growth was recorded twice a week with a caliper-like instrument. Tumor volume was calculated according to the formula volume = (width2 × length)/2. The formation of metastasis was observed and assessed by bioluminescence imaging using a Xenogen IVIS 200 Imaging System (Caliper Life Sciences, Hopkinton, MA, UAS) at week 6. The mice were sacrificed at 6 weeks considering animal welfare and the final volume and weight of tumor tissues were determined. All in vivo experiments were reviewed and approved by the Animal Ethics Committee of TMUCIH and were performed according to the guidelines for the welfare and use of animals in cancer research [26] and national law.

### Statistical analysis

Data are presented as mean ± standard deviation. The Student’s *t*-test (2-tailed) was used to determine the differences between the experimental and control groups. The level of significance was set to *P* < 0.05. Kaplan-Meier survival curves and the log-rank test were used to evaluate patients of breast cancer with different miR-190 expression. Spearman’s correlation was used to test the significance of association between miR-190 and SMAD2 expression. All calculations were performed with the SPSS for Windows statistical software package (SPSS Inc., Chicago, IL, USA).

## Results

### Down-regulation of miR-190 is required for cell invasion through regulating EMT

**Fig. 1:**
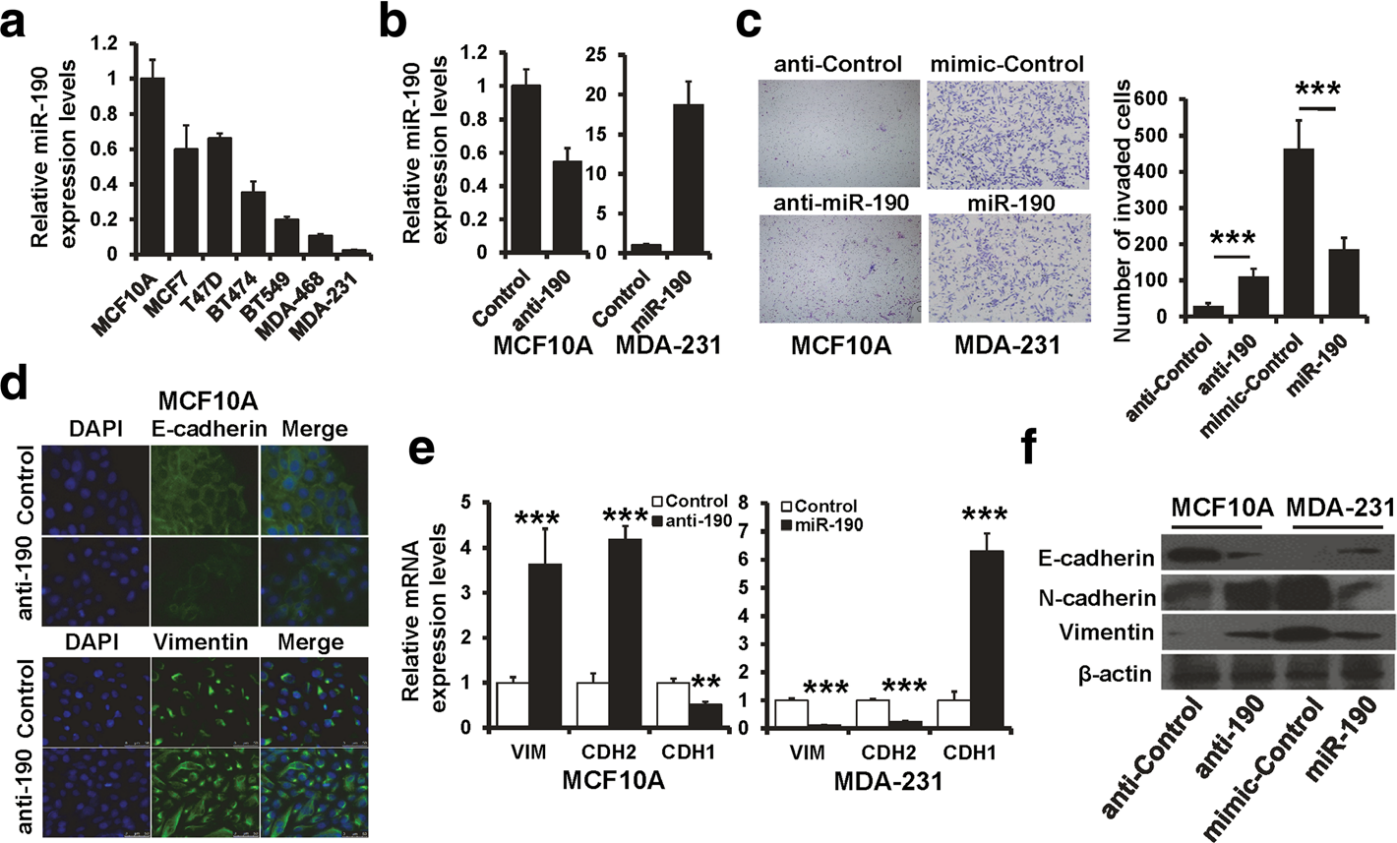
miR-190 suppresses breast cancer invasion and EMT phenotype. **a** The miR-190 expression in breast cancer cell lines as determined by RT-qPCR. **b** The miR-190 expression in MCF10A cells transfected with miR-190 inhibitor and MDA-MB-231 cells transfected with miR-190 mimic, as determined by RT-qPCR. **c** Transwell invasion of cells as in (**b**). **d** Immunofluorescence analyses of EMT markers in MCF10A cells. **e** and **f** The mRNA (**e**) and protein (**f**) expression of EMT markers in cells as in (**b**) were detected by RT-qPCR and western blotting. ***P < 0.001, **P < 0.01

To investigate the role of miR-190 in breast cancer progression, we determined the expression of miR-190 in breast cancer cell lines (MCF7, T47D, BT474, BT549, MDA-MB-468, and MDA-MB-231) and normal breast epithelial cell line (MCF10A) by RT-qPCR. The expression of miR-190 was down-regulated in all breast cancer cell lines as compared to MCF10A cells (Fig. 1a). Next, we investigated the influence of miR-190 on cell proliferation and invasion by transfecting MCF10A and MDA-MB-231 cells with miR-190 inhibitor or mimic (Fig. 1b). MTT and colony formation assays indicated that miR-190 expression did not alter the cell proliferation in MCF10A and MDA-MB-231 cells (Additional file 1: Figure S1A and S1B). However, overexpression of miR-190 reduced cell invasion in MDA-MB-231 cells, while its inhibition enhanced cell invasion in MCF10A cells compared to control cells, as determined by transwell assay (Fig. 1c). EMT plays a pivotal role during malignant tumor progression and metastasis, and thus, we next determined whether or not miR-190 affects breast cancer progression through regulation of EMT. We examined both epithelial and mesenchymal marker expression by immunofluorescence (Fig. 1d), RT-PCR (Fig. 1e), and western blotting (Fig. 1f). As can be seen, the miR-190-depleted MCF10A cells showed significant down-regulation of E-cadherin, while the mesenchymal markers, vimentin and N-cadherin, were dramatically up-regulated (Fig. 1d, e left, and 1F left). Furthermore, we observed an increased E-cadherin expression and decreased vimentin and N-cadherin expression in miR-190-expressing MDA-MB-231 cells (Fig. 1e right and 1f right). Similar results were also observed in BT549 cells (Additional file 1: Figure S3A–S3F). Together, these results suggest that miR-190 suppresses EMT and breast cancer progression.

### miR-190 inhibits breast cancer metastasis in vivo

**Fig. 2:**
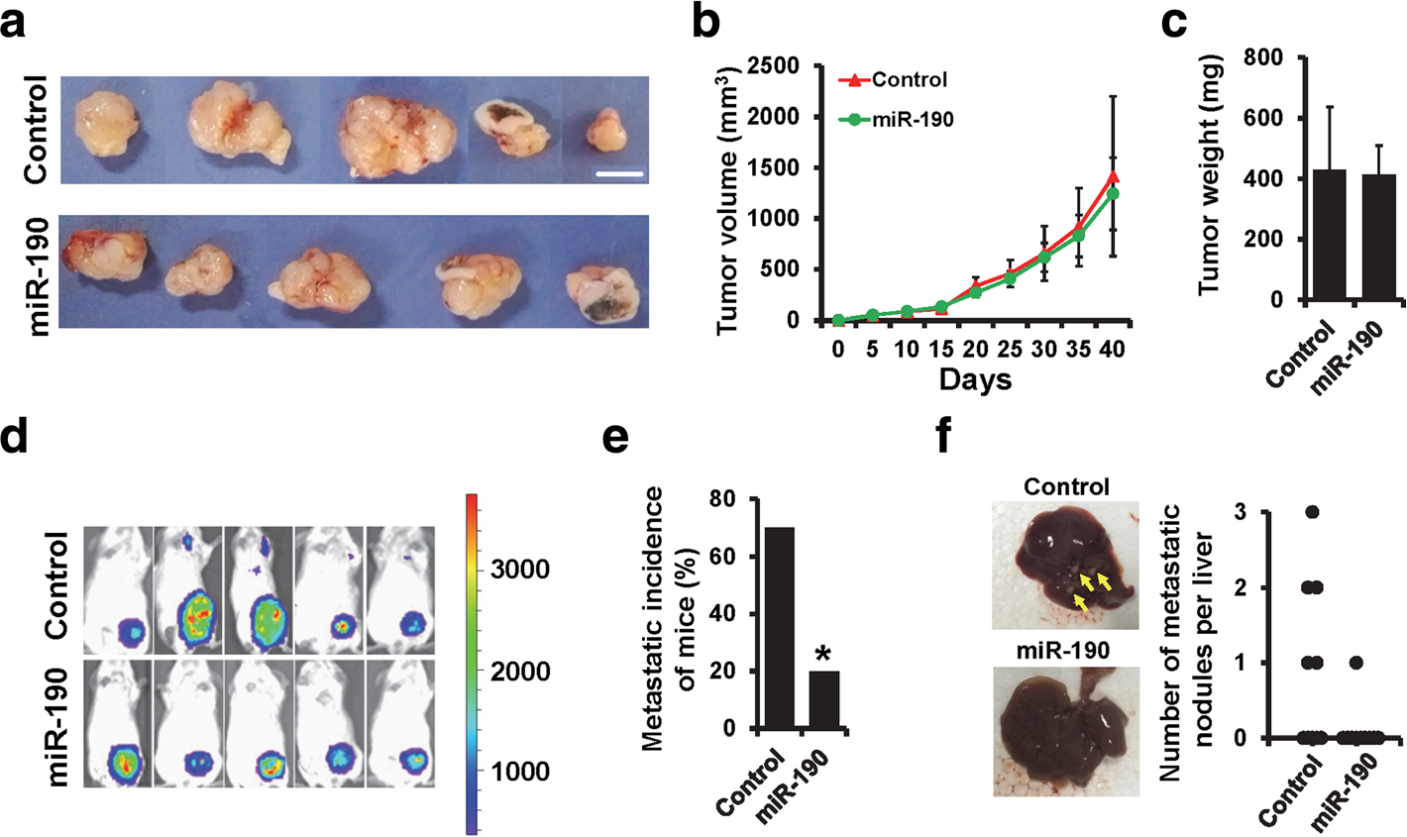
miR-190 suppresses breast cancer metastasis in vivo. **a** Representative photos of the tumors formed by MDA-MB-231-luc-miR-190 or control cells at harvest time. **b** Tumor volume of xenograft mice injected with MDA-MB-231-luc-miR-190 or control cells at the indicated times. **c** The weights of tumors formed by MDA-MB-231-luc-miR-190 or control cells at harvest time. **d** Bioluminescence images of tumors formed by MDA-MB-231-luc-miR-190 or control cells. **e** Metastatic incidence of xenograft mice injected with MDA-MB-231-luc-miR-190 or control cells. **f** Representative photos (left) and counts (right) of visible metastatic nodules in the liver of euthanized xenograft mice injected with MDA-MB-231-luc-miR-190 or control cells. Scale bar, 1 cm. *P < 0.05

To confirm the role of miR-190 in breast cancer metastasis in vivo, we generated stable miR-190-expressing cells by lentiviral infection of MDA-MB-231-luc cells (Additional file 1: Figure S2A). The MTT and colony formation assays also indicated that overexpression of miR-190 did not affect MDA-MB-231-luc cell proliferation (Additional file 1: Figure S2B and 2C). However, the ability of cell invasion was significantly decreased in miR-190-expressing MDA-MB-231-luc cells (Additional file 1: Figure S2D). MDA-MB-231-luc cells stably expressing miR-190 (231 L-miR-190) and control-transfected cells (231 L-control) were implanted into the mammary fat pads of nude mice, and tumor growth and metastasis were quantified. Overexpression of miR-190 in MDA-MB-231-luc cells did not affect the tumor growth in vivo (Fig. 2a–c). We next performed bioluminescence imaging to monitor metastasis in vivo using an Xenogen IVIS system. Consistent with the cell proliferation experiments in vitro, there was no significant difference in the tumor volume between 231 L-miR-190 and 231 L-control cells; however, 231 L-miR-190 mice suffered significantly less metastatic dissemination than the 231 L-control mice (Fig. 2d). The data revealed that 70% of 231 L-control mice (7/10) suffered metastasis, whereas only 20% of the 231 L-miR-190 mice (2/10) suffered metastasis (Fig. 2e). Visible liver metastatic nodules were observed in half of the 231 L-control mice (5/10), whereas only one was observed in the liver of 231 L-miR-190 mice (Fig. 2f). Similar results were also observed in BT549 cells (Additional file 1: Figure S3G-S3I). Together, these results indicate that miR-190 suppresses breast cancer metastasis in vivo.

### miR-190 reverses TGF-β-induced EMT

**Fig. 3:**
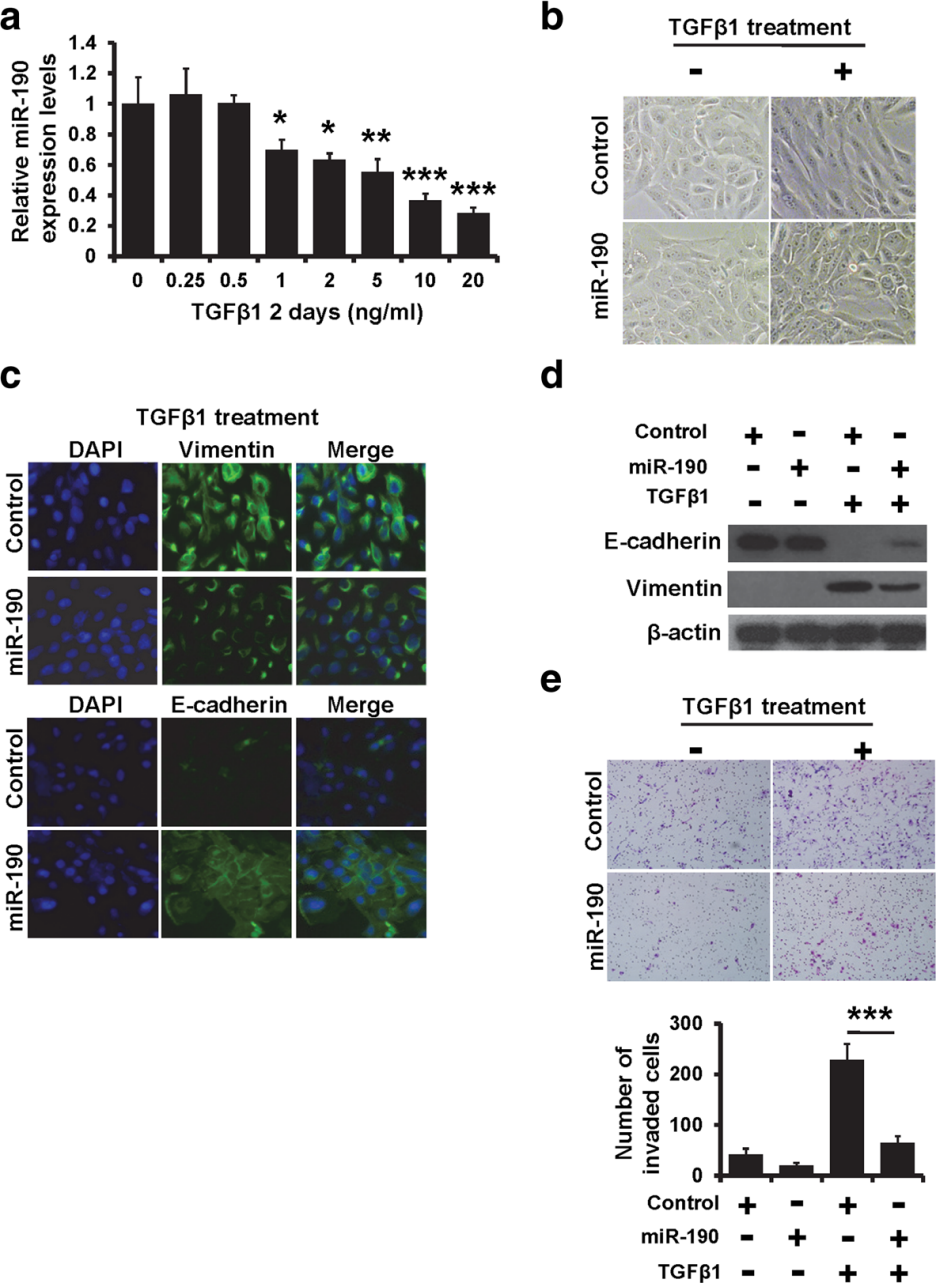
miR-190 down-regulation is required for TGF-β-induced EMT. **a** The miR-190 expression in MCF10A cells after treatment with TGF-β1 at indicated concentration. **b** Morphological photos of miR-190-overexpressing MCF10A or control cells with or without TGF-β1 treatment. **c** The expression of EMT markers in miR-190-overexpressing MCF10A or control cells after TGF-β1 treatment, as determined by immunofluorescence staining. **d** The expression of EMT markers in miR-190-overexpressing MCF10A or control cells after TGF-β1 treatment, as determined by western blotting. **e** Transwell invasion of cells as in (**b**). ***P < 0.001, **P < 0.01, *P < 0.05

TGF-β is a major inducer of EMT in development, carcinogenesis, and fibrosis. TGF-β1 was an inducer of EMT in normal mammary epithelial cells [11, 27]. Here, we showed that miR-190 expression was decreased after addition of TGF-β1 to the cell culture medium for 2 days (Fig. 3a). After treatment with TGF-β1, MCF10A cells displayed an elongated fibroblast-like morphology, whereas miR-190-expressing MCF10A cells retained their cobblestone-like morphology (Fig. 3b). Consistent with morphological changes, immunofluorescence staining revealed that miR-190-expressing MCF10A cells maintained the expression of the epithelial marker E-cadherin at the cell membrane, and expression of the mesenchymal marker vimentin was decreased compared to that observed in TGF-β1-treated control cells (Fig. 3c). Failure to lose epithelial and gain mesenchymal marker expression in TGF-β1-treated miR-190-expressing MCF10A cells was further confirmed by western blotting (Fig. 3d). As shown in Fig. 3e, the invasive ability of MCF10A cells was dramatically increased after treatment with TGF-β1. In contrast, miR-190-expressing MCF10A cells decreased the invasive ability of TGF-β1-treated MCF10A cells (Fig. 3e). These results suggest that miR-190 is involved in TGF-β-induced EMT.

### miR-190 blocks TGF-β signaling by targeting SMAD2

**Fig. 4:**
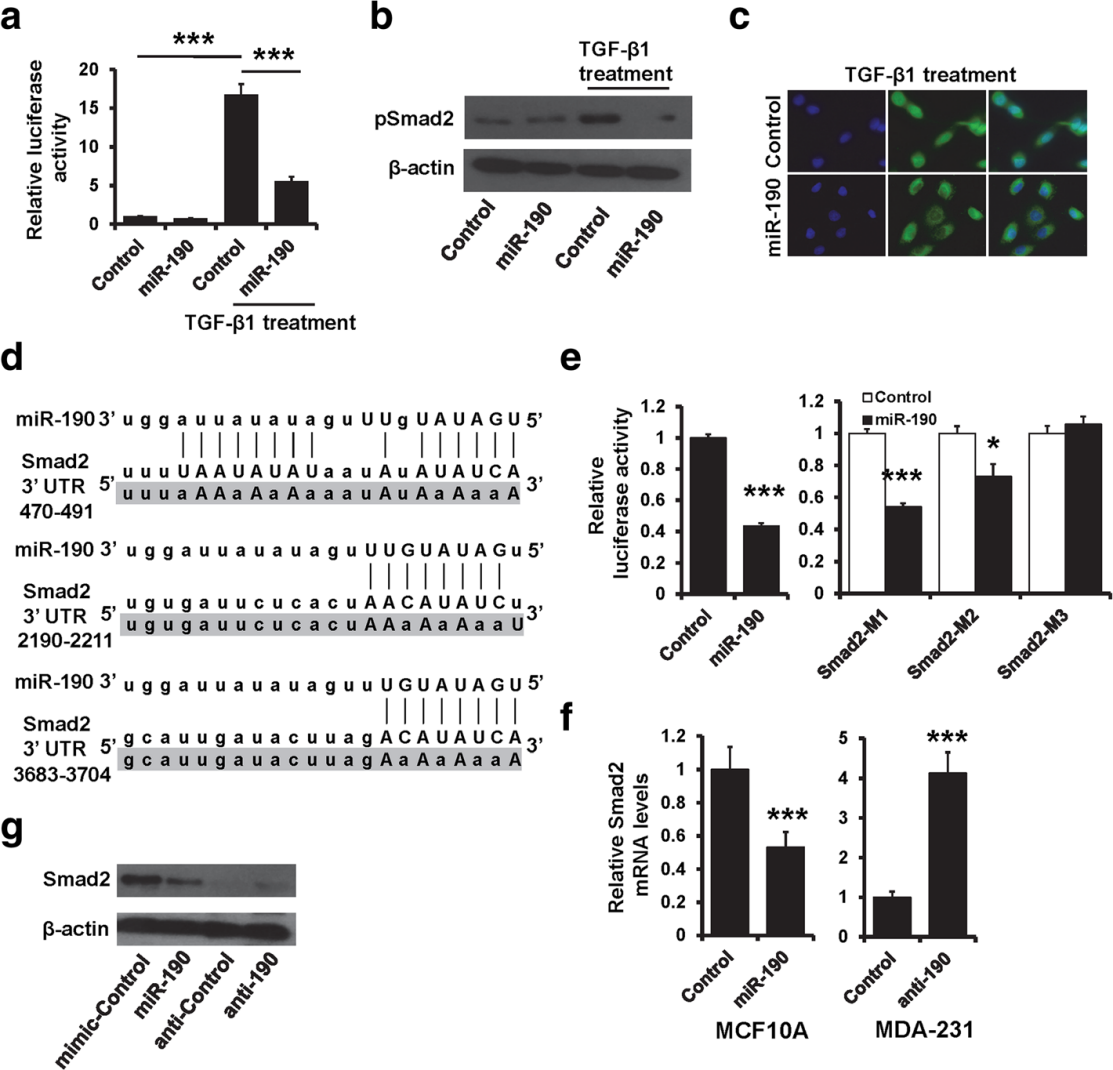
miR-190 regulates TGF-β signaling by targeting SMAD2. **a** Luciferase reporter analysis of TGF-β signaling activity in miR-190-overexpressing MDA-MB-231 or control cells with or without TGF-β1 treatment. **b** The expression of pSMAD2 in cells as in (**a**). **c** Localization of SMAD2 in miR-190-overexpressing MDA-MB-231 or control cells with TGF-β1 treatment, as determined by immunofluorescence staining. **d** The predicted binding of miR-190 with SMAD2 3′-UTR. **e** Dual luciferase reporter system analysis was performed to validate the miR-190 target, SMAD2. **f** The miR-190 expression in indicated cells as determined by RT-qPCR. **g** The expression of SMAD2 in indicated cells as determined by western blotting. ***P < 0.001, *P < 0.05

To further explore the role of miR-190 in TGF-β signaling, we detected the luciferase activity of SMAD reporter in miR-190-expressing MDA-MB-231 and control cells with or without TGF-β1 treatment. The luciferase activity was dramatically increased in TGF-β1-treated cells, but this effect was reversed in miR-190-expressing MDA-MB-231 cells (Fig. 4a). The expression of pSMAD2 and nuclear localization of SMAD2 was also decreased in TGF-β1-treated MDA-MB-231 cells after transfection with miR-190 mimic (Fig. 4b and c). These results indicated that miR-190 blocks TGF-β signaling. The target prediction program, TargetScan, was applied to identify SMAD2 as a putative miR-190 target (Fig. 4d). To further confirm this regulation, SMAD2 3′-UTR and its mutant containing the putative miR-190 binding sties were cloned into the downstream luciferase ORF (Fig. 4d). As compared to control cells, the luciferase activity was significantly decreased in miR-190-expressing MDA-MB-231 cells with inhibition rates of 60% (Fig. 4e left). These effects were abolished in mutated SMAD2 3′-UTR, in which the binding sites for miR-190 were inactivated (SMAD2-M3) by site-directed mutagenesis (Fig. 4e right). The expression of SMAD2 was decreased in miR-190-expressing MDA-MB-231 cells and was increased in miR-190-depleted MCF10A cells compared with that of control cells, as determined by RT-qPCR (Fig. 4f) and western blotting (Fig. 4g). Together, these results indicated that miR-190 blocks TGF-β signaling by targeting SMAD2.

### ZEB1 transcriptionally suppresses miR-190 expression

**Fig. 5:**
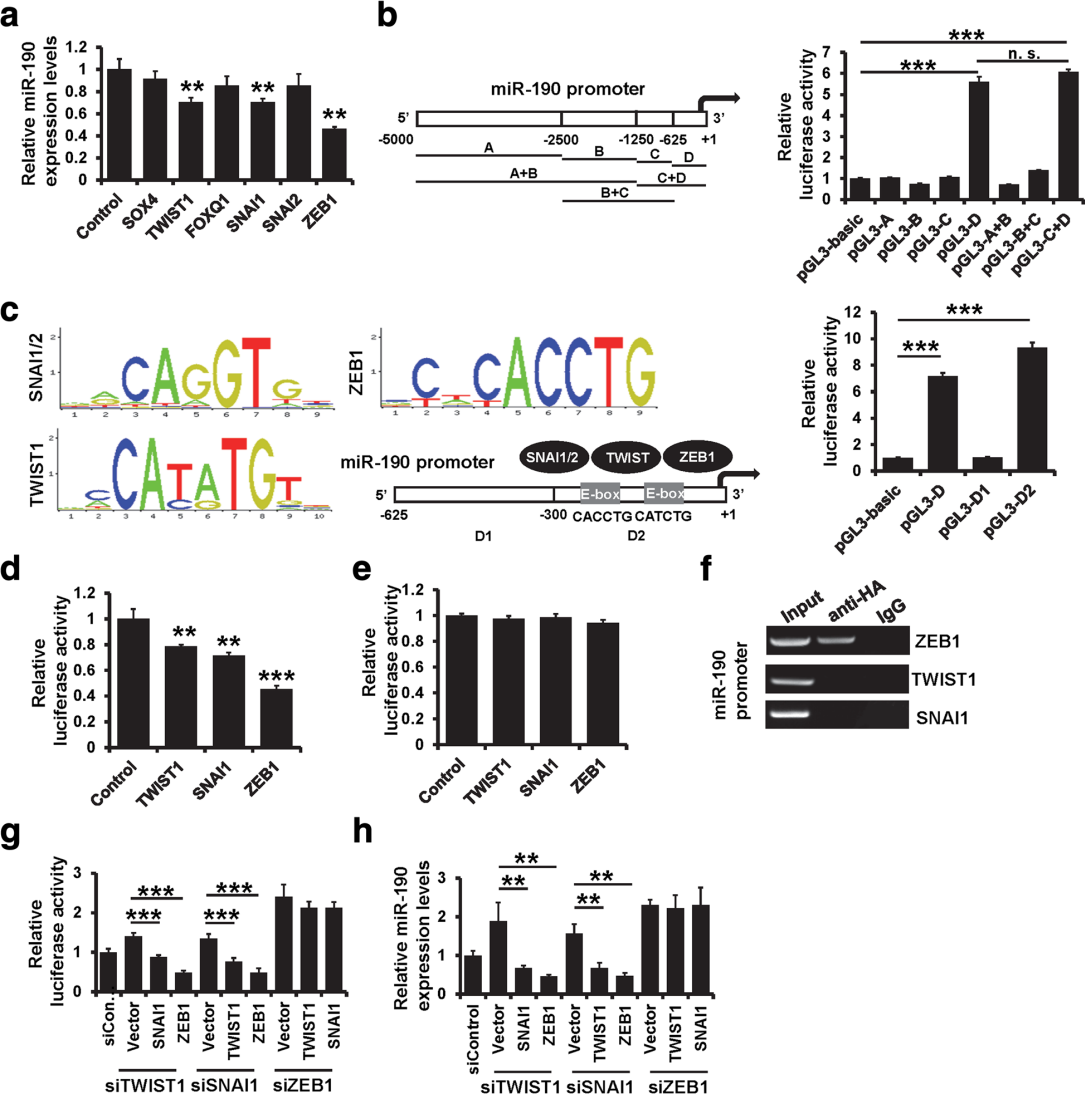
ZEB1 transcriptionally suppresses miR-190. **a** The expression of miR-190 in MCF10A cells transfected with EMT-related transcription factors or control cells, as determined by RT-qPCR. **b** Promoter analysis of miR-190. Different putative miR-190 promoter region (left) were inserted in front of the pGL3-basic luciferase reporter and luciferase activity was measured. **c** Determination of the core promoter of miR-190. Two E-boxes were located on the core promoter of miR-190 (− 300 to + 1) and luciferase activity was measured. **d** miR-190 promoter activity was measured in MCF10A cells transfected with TWIST1, SNAI1, or ZEB1. **e** Mutation of E-box sequence prevents ZEB1 from inhibiting the miR-190 promoter. **f** Interaction between TWIST1, SNAI1, or ZEB1 and the miR-190 promoter sequence in MCF10A cells, as determined by ChIP assay. **g** Luciferase activity of miR-190 promoter in indicated cells. **h** The miR-190 expression in indicated cells as determined by RT-qPCR. ***P < 0.001, **P < 0.01

**Fig. 6:**
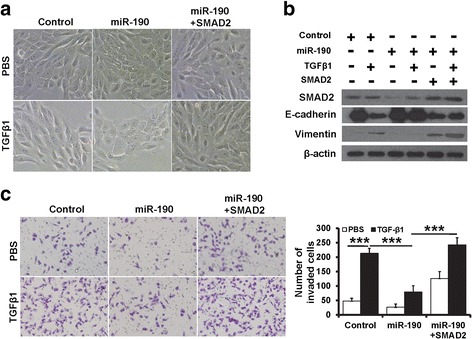
SMAD2 rescues the effect of miR-190 in TGF-β-induced EMT. **a** Morphological photos of miR-190-overexpressing MCF10A or control cells with or without TGF-β1 treatment. **b** The expression of EMT markers and SMAD2 in cells as in (**a**), as determined by western blotting. **c** Transwell invasion of cells as in (**a**). ***P < 0.001

EMT is mainly regulated by a restricted number of transcription factors (TFs), including ZEB, SNAI, TWIST, SOX, and FOX families. We next investigated whether these EMT-TFs regulate the expression of miR-190. We detected the expression of miR-190 in MCF10A cells expressing HA-tagged SOX4, TWIST1, FOXQ1, SNAI1, SNAI2, and ZEB1. We observed that the expression of miR-190 was decreased in TWIST1-, SNAI1-, SNAI2-, and ZEB1-overexpressing MCF10A cells (Fig. 5a). All these factors bind to E-boxes in the regulatory region of target genes and act as transcriptional repressors (E-TFs). Next, we tried to identify the core promoter region of miR-190 by cloning several deletions of the region starting 5000 bp upstream of the miR-190 promoter into the pGL3-basic reporter (Fig. 5b left). We found that pGL3-(D) and pGL3-(C + D) resulted in high luciferase activity (Fig. 5b right). We next examined the promoter sequence of miR-190 and, interestingly, found two E-boxes on the miR-190 promoter region (Fig. 5c). Based on these results, we hypothesized that miR-190 transcription is regulated by E-TFs. To confirm this, we separated the D region into two parts, D-2 containing the E-box and D-1 lacking the E-box, and linked each of them to the reporter gene and measured their luciferase activity. As shown in Fig. 6c, the cells transfected with D-2 showed high luciferase activity, while those transfected with D-1 showed no luciferase activity, suggesting that the D-2 region is the core promoter region of miR-190.

To examine whether E-TFs regulate miR-190 promoter (− 300 to + 1) activity, we transfected the E-TFs into MCF10A cells. As shown in Fig. 5d, the luciferase activity was decreased in all the E-TF-transfected cells. We next constructed a mutant clone in which the E-box sequence was mutated from CACCTG to TTTTTT, and used it to transfect the MCF10A cells. As shown in Fig. 5e, this mutant showed no altered luciferase expression after transfection with E-TFs. We then used ChIP assay to immunoprecipitate HA and used primers to amplify the D-2 region of the miR-190 promoter region. As shown in Fig. 5f, only ZEB1 was able to bind directly to the miR-190 promoter region. To further confirm whether only ZEB1 transcriptionally suppresses miR-190, we transfected two E-TF expression plasmids and siRNAs targeting the other E-TFs. As shown in Fig. 5g, the luciferase activity of miR-190 promoter was no longer affected by TWIST1 or SNAI1 in ZEB1-depleted cells. The expression of miR-190 was also not affected by TWIST1 or SNAI1 in ZEB1-depleted cells (Fig. 5h). Together, these results indicated that ZEB1 transcriptionally suppresses miR-190 expression.

### miR-190 inhibits EMT and invasion by suppressing SMAD2

To corroborate that SMAD2 mediates the role of miR-190 in TGF-β-induced responses in breast cancer cells, we transfected HA-SMAD2 plasmid into MCF10A cells in addition to TGF-β stimulation and miR-190 overexpression. While miR-190 overexpression led to SMAD2 suppression in TGF-β-treated cells and abolished TGF-β-induced EMT phenotypes, transient transfection of HA-SMAD2 restored cellular responses to TGF-β. There was no significant change in the expression of SMAD2 and EMT markers and cellular morphology after TGF-β stimulation, despite the overexpression of miR-190 (Fig. 6a and b), suggesting that SMAD2 inhibition mediates the effects of miR-190 overexpression on breast cancer cell EMT. Furthermore, SMAD2 overexpression abolished the effects of miR-190 on MCF10A cell invasion. With forced expression of SMAD2, miR-190 was no longer able to decrease MCF10A cell invasion after TGF-β stimulation (Fig. 6c). Thus, these results indicated that miR-190 inhibits EMT and breast cancer invasion by suppressing SMAD2.

### miR-190 predicts favorable outcome and inversely correlates with SMAD2 in clinical samples

**Fig. 7:**
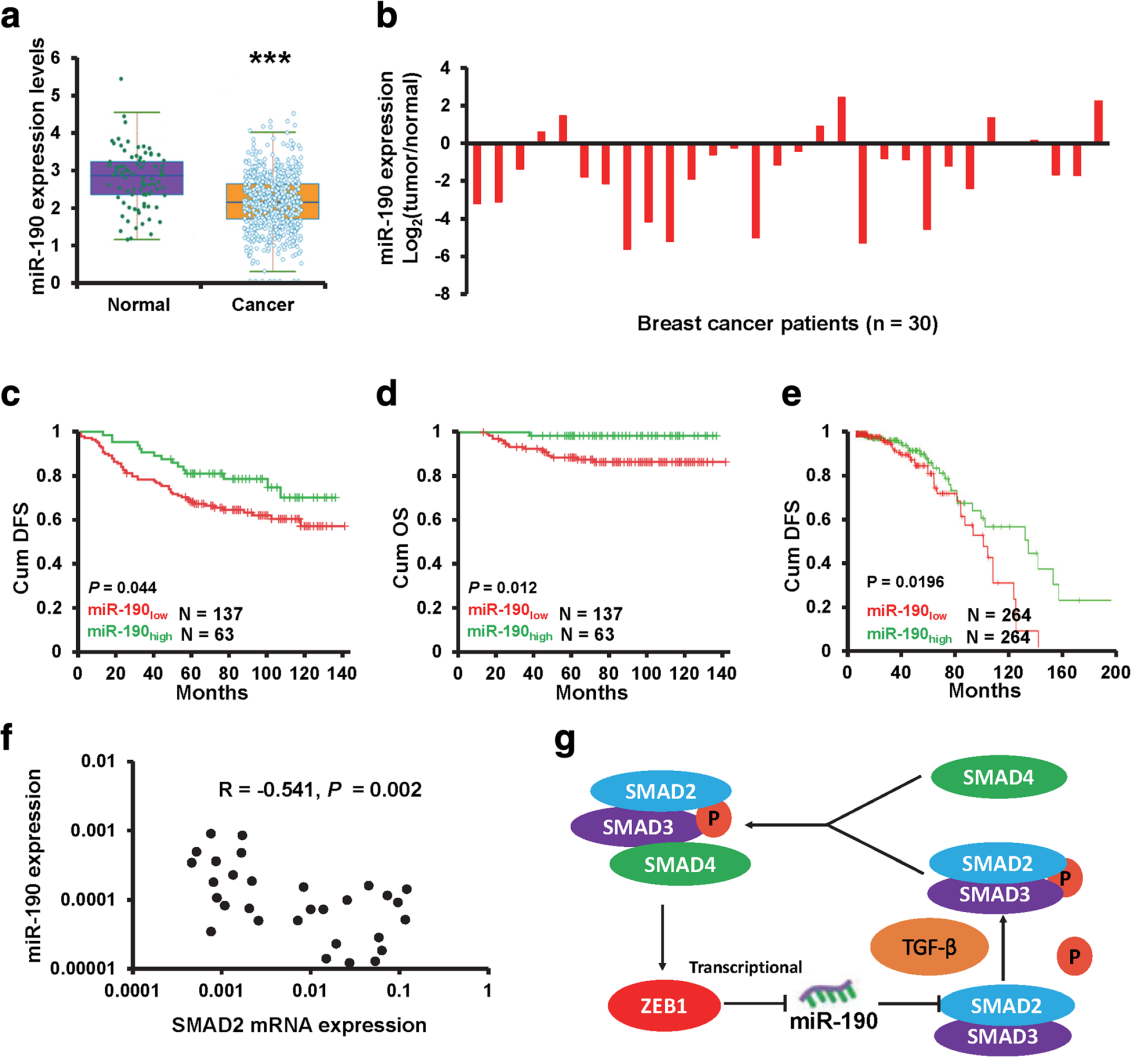
miR-190 is down-regulated and negatively correlated with SMAD2 expression in breast cancer tissues. **a** The miR-190 expression in TCGA breast invasive carcinoma and normal tissues. **b** The miR-190 expression in primary breast cancer tissues and paired normal breast tissues. **c** and **d** Kaplan-Meier analysis of the DFS (**c**) and OS (**d**) in patients with different miR-190 expression levels, as determined by RT-qPCR. **e** Kaplan-Meier analysis of the DFS in patients with different miR-190 expression levels, as determined using TCGA database. **f** The relationship between miR-190 and SMAD mRNA expression, as determined by RT-qPCR. **g** A model for the role of miR-190 in breast cancer metastasis. ***P < 0.001

We finally examined the clinical relevance of miR-190 expression in breast cancer progression. We analyzed the expression of miR-190 in clinical breast cancer samples of The Cancer Genome Atlas (TCGA) database and found that miR-190 expression was significantly lower in breast cancer tissues than in normal tissues (Fig. 7a). Furthermore, miR-190 expression was also determined in 30 samples of primary breast cancer tissues and paired adjacent normal breast tissues by RT-qPCR. We observed down-regulation of miR-190 in most of the cases (Fig. 7b). More importantly, we compared the disease-free survival (DFS) and overall survival (OS) of breast cancer patients with different levels of miR-190 expression and found that patients with high miR-190 expression had a significantly favorable DFS (Fig. 7c) and OS (Fig. 7d) compared to those with low miR-190 expression. A similar result was also observed in TCGA database (Fig. 7e). To further investigate the clinical relationship between SMAD2 and miR-190, we examined the expression of SMAD2 in 30 specimens of primary breast cancer tissues by RT-qPCR. As shown in Fig. 7f, the expression of SMAD2 exhibited a significant relationship with miR-190 expression. Collectively, our data support the conclusion that miR-190 targets SMAD2 to suppress TGF-β signaling in breast cancer cell lines and human breast cancer specimens.

## Discussion

In the present study, we identified miR-190 as an EMT inhibitor and a tumor suppressor, and validated that miR-190 is decreased in clinical breast cancer tissues and correlates with survival in patients with breast cancer. Overexpression of miR-190 inhibits TGF-β signaling via down-regulation of SMAD2 expression and suppresses breast cancer metastasis both in vitro and in vivo. Furthermore, miR-190 is a direct transcriptional target of ZEB1 and mediates TGF-β-induced EMT. Therefore, our results revealed a novel mechanism for constitutive TGF-β activation in breast cancer and demonstrated that miR-190 functions as a tumor-suppressive miRNA in breast cancer.

Dysregulation of miRNAs is involved in almost every cellular process during tumorigenesis and progression, including differentiation, proliferation, apoptosis, migration, invasion, angiogenesis, and EMT. It has been indicated that miR-190 expression is decreased in aggressive neuroblastoma, prostate cancer, and hepatocellular carcinoma, and overexpression of miR-190 leads to inhibition of tumor growth and metastasis [20, 28, 29]. miR-190 is also involved in estrogen receptor signaling, causing inhibition of breast cancer metastasis [22]. However, miR-190 is up-regulated in gastric cancer tissues and contributes to gastric cancer progression [23]. Consistent with most previous studies, we provided evidence that miR-190 is down-regulated in breast cancer tissues and correlates with better survival in patients with breast cancer. Furthermore, we observed that miR-190 suppresses breast cancer metastasis both in vitro and in vivo. However, tumor cell proliferation was not significantly affected by altered expression of miR-190. This is in line with observations from other experimental models of tumor proliferation in osteosarcoma and glioblastoma in which tumor cell proliferation is balanced by cell death, which results in persistence of a constant tumor size [20]. Our study reveals that miR-190 functions as a tumor suppressor in breast cancer metastasis.

Metastasis is a multistep process where tumor cells leave the primary tumor, disseminate to distant sites, and form secondary tumors [12]. EMT has been shown to play pivotal roles in these steps to promote metastasis. During the EMT process, epithelial cells lose their characteristics and acquire an invasive mesenchymal phenotype. The decreased expression of epithelial marker E-cadherin and increased expression of mesenchymal markers, vimentin and N-cadherin, are often observed when EMT occurs. Similar to the observation in hepatocellular carcinoma [28], we found that miR-190 induced the expression of E-cadherin and reduced the expression of vimentin and N-cadherin in breast cancer cells, whereas inhibition of endogenous miR-190 by its inhibitors yielded the opposite effects, suggesting that miR-190 is an EMT suppressor in breast cancer. TGF-β is a key driver of EMT and plays an important role in cancer metastasis. It represses E-cadherin expression and induces the expression of vimentin and N-cadherin, thus promoting EMT and cancer metastasis [12]. TGF-β signaling is initiated with ligand-induced oligomerization of serine/threonine receptor kinases and phosphorylation of the cytoplasmic signaling molecules, SMAD2 and SMAD3 [30]. Inhibition of TGF-β/SMAD2 has been shown to reverse the EMT-phenotype and suppress breast cancer metastasis [31–33]. Here, we demonstrated that miR-190 down-regulation is required for TGF-β-induced EMT, and SMAD2 is a direct target of miR-190. These results indicated that miR-190 suppresses breast cancer metastasis and EMT phenotype by targeting SMAD2.

ZEB1 is a crucial member of the zinc finger homeodomain transcription factor family involved in the regulation of cell fate determination and differentiation [34]. The zinc finger structures are highly conserved and allow DNA binding at the E-box in the promoter region of target genes, such as E-cadherin [35]. Here, we found that ZEB1 functions as a transcriptional repressor, which depends on the E-box binding sites on the miR-190 promoter region. Aberrant expression of ZEB1 has been observed in many human cancers, including breast cancer [36–39]. ZEB1 expression negatively correlates with E-cadherin expression and is associated with advanced diseases or metastasis, which indicates the relevance of ZEB1 induction of EMT and cancer development [40]. The expression of ZEB1 is regulated by multiple signaling pathways, such as nuclear factor-κB, WNT, TGF-β, and miRNA. The important regulatory link between ZEB1 and miRNAs has been identified by several groups. The miR-200 family has been highly implicated in the regulation of EMT. Repression of ZEB1 by miR-200 family results in an increased expression of epithelial markers and a decreased expression of mesenchymal markers [41, 42]. Our results showed that ZEB1 binds to the miR-190 promoter and transcriptionally suppresses miR-190 expression, suggesting that ZEB1 promotes breast cancer metastasis and EMT phenotype through inhibition of miR-190 expression.

## Conclusions

In summary, we demonstrated that miR-190 is a tumor suppressor in breast cancer and also an independent predictor in patients with breast cancer. miR-190 suppresses breast cancer metastasis and EMT phenotype by targeting SMAD2. Furthermore, miR-190 is a transcriptional target of ZEB1. Based on the findings from this study and others, we propose a model that highlights the role of miR-190 in regulating TGF-β signaling during breast cancer metastasis (Fig. 7g). The uncovering of this ZEB1-miR-190-SMAD axis will extend our comprehension of TGF-β network complexity and miR-190 as a new option to target TGF-β signaling for breast cancer intervention.

## Additional file

Additional file 1: Supplementary Materials and Methods. **Figure S1.** miR-190 does not affect cell proliferation in breast cancer. **Figure S2.** miR-190 promotes cell invasion but does not affect cell proliferation in MDA-MB-231-luc cells. **Figure S3.** miR-190 suppresses breast cancer metastasis and EMT phenotype in BT549 cells. **Table S1.** Primers used for RT-qPCR. (DOCX 2080 kb)
