# Supplementary material for: miR-190 suppresses breast cancer metastasis by regulation of TGF-β-induced epithelial–mesenchymal transition
Source: Mol Cancer. 2018 Mar 6;17:70. doi: 10.1186/s12943-018-0818-9 (PMC5838994; doi:10.1186/s12943-018-0818-9)
Supplement: Supplementary file 1 — Supplementary Materials and Methods. Figure S1. miR-190 does not affect cell proliferation in breast cancer. Figure S2. miR-190 promotes cell invasion but does not affect cell proliferation in MDA-MB-231-luc cells. Figure S3. miR-190 suppresses breast cancer metastasis and EMT phenotype in BT549 cells. Table S1. Primers used for RT-qPCR. (DOCX 2080 kb) [file 12943_2018_818_MOESM1_ESM.docx]

**Supplemental data**

**Supplementary Materials and Methods**

**Antibodies and reagents**

Antibodies against N-cadherin, E-cadherin, vimentin, HA (Santa Cruz Biotechnology, Santa Cruz, CA, USA), TWIST1, SNAI1, ZEB1 (Abcam, Cambridge, MA, USA), SMAD2, pSMAD2, and β-actin (Cell Signaling Technology, Beverly, MA, USA) were used. Recombinant human TGF-β1 and EGF were purchased from R&D Systems (Redmond, WA, USA). Cholera toxin, hydrocortisone, insulin, and TGF-βRI inhibitor SB431542 were purchased from Sigma-Aldrich (St Louis, MO, USA).

**Plasmid, miRNA, and siRNAs**

The miR-190 mimic, miR-190 inhibitor, or the appropriate scrambled controls were purchased from RiboBio (Shanghai, China). The TWIST1, SNAI1, SNAI2, and ZEB1 gene-specific siRNAs, and non-specific control siRNA were also purchased from RiboBio. pCMV5 SMAD2-HA (Addgene; plasmid # 14930). The SMAD2 3ʹ-UTR containing miR-190 binding sites were amplified and cloned into psiCHECK2 vector (Promega, Madison, WI, USA) to generate Luc-SMAD2. Site-directed mutagenesis was performed using the Site-Directed Mutagenesis Kit (TransGene, Beijing, China) to generate the SMAD2 3ʹ-UTRmut reporter vector (SMAD2M1/2/3). The miR-190 promoter region (–300 to +1) and the E-box mutated fragments were cloned into pGL3-Basic vector (Promega; miR-190pW and miR-190pM). All constructs were confirmed by sequencing.

**Proliferation and invasion assays**

Both MTT and plate colony formation assays were used to evaluate the ability of cell proliferation. For MTT assay, 24 h after transfection, cells were seeded into 96-well plates at a density of 5 × 10^3^ cells/per well. After incubation for the indicated time, cells were incubated with 10 μL MTT (0.5 mg/mL; Sigma-Aldrich) at 37°C for 4 h. The medium was then removed, and precipitated Formosan was dissolved in 150 μL DMSO. The absorbance at 570 nm was detected using a micro-plate auto-reader (Bio-Rad, Richmond, CA, USA). For plate colony formation assay, 24 h after transfection, cells were seeded in 6-well plates at a density of 500 cells/per well. After about 3 weeks, the colonies obtained were washed with phosphate buffered saline (PBS) and fixed with 10% formalin for 15 min at room temperature and then washed with PBS, followed by staining with hematoxylin. The number of colonies were counted and compared with control.

The invasive abilities of breast cancer cells *in vitro* were evaluated by Matrigel-coated Transwell (BD Biosciences, San Diego, CA, USA). Briefly, 5 × 10^4^ cells in 500 μL serum-free medium were added to the upper chamber, and medium containing 20% FBS was added into the lower chamber. Twenty-four hours later, the migrant cells that had attached to the lower surface were fixed with 20% methanol and stained for 20 min with crystal violet. The membranes were then carved and embedded under coverslips with the cells on the top. The number of migrating cells was counted under a microscope in five predetermined fields.

**Western blotting**

Cells were lysed in protein lysis buffer [20 mM Tris-HCl (pH 7.4), 5 mM EDTA, 1% Triton X-100, 150 mM NaCl, and 1% DTT] containing a protease inhibitor cocktail tablet (Roche Molecular Biochemicals, Indianapolis, IN, USA). Protein lysates were resolved by sodium dodecyl sulfate-polyacrylamide gel electrophoresis, transferred to polyvinylidene fluoride membranes (Millipore, Bedford, MA, USA), detected with primary antibody overnight at 4°C, and then incubated with horseradish peroxidase-conjugated secondary antibodies. The blots were visualized with ECL reagent (Millipore).

**Immunofluorescence**

Cells were seeded onto glass coverslips in 24-well plates, washed with PBS, fixed in 4% formaldehyde solution for 30 min, and then permeabilized with 0.2% Triton X-100/PBS for 15 min. Cells were blocked with 2% bovine serum albumin in PBS for 30 min. Coverslips were incubated with primary antibodies overnight at 4°C, followed by incubation with FITC-/TRITC-conjugated secondary antibodies for 1 h at room temperature, and then stained with DAPI. Finally, coverslips were observed under a fluorescence microscope.

**Supplemental figure legends**

**
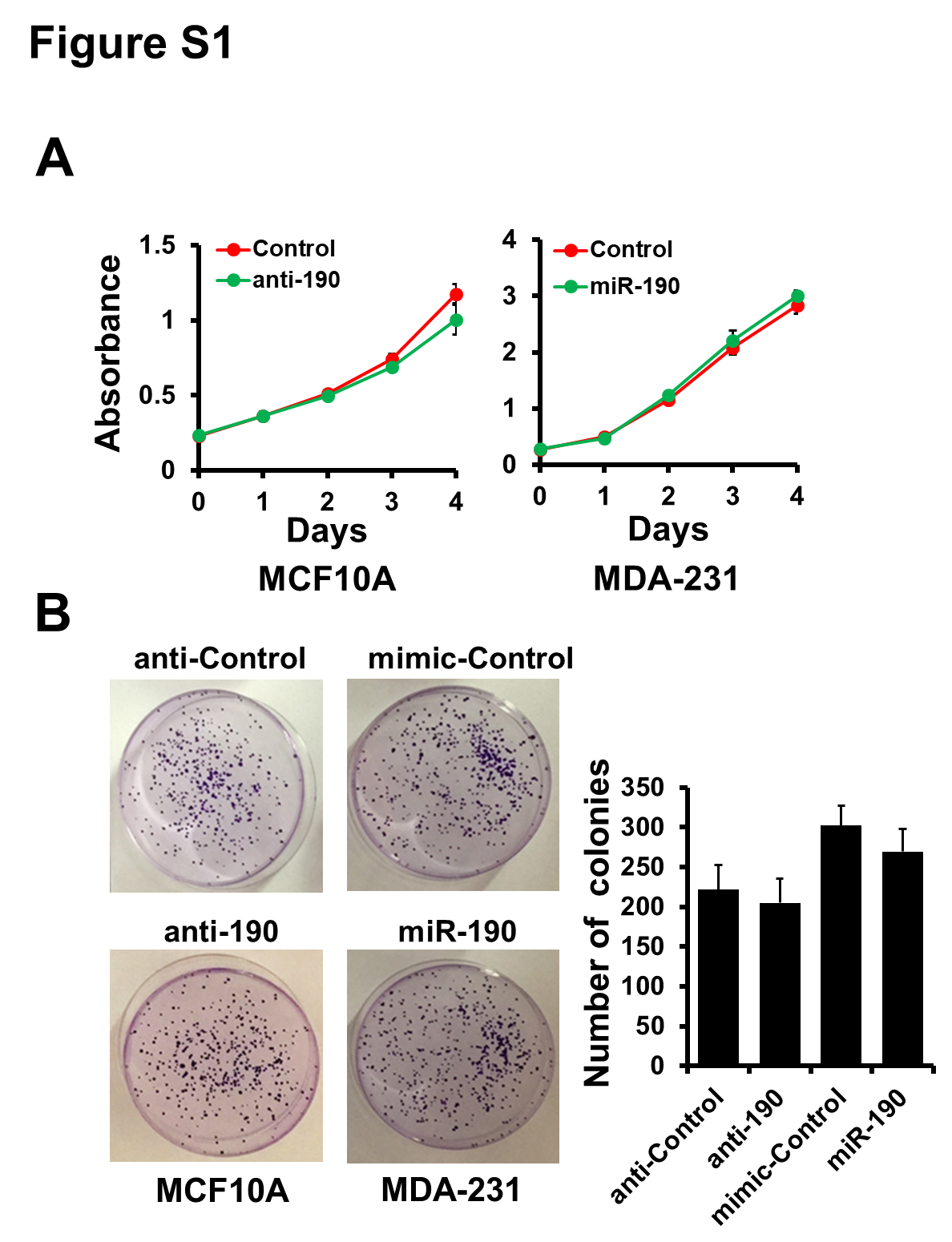
**

**Figure S1. miR-190 does not affect cell proliferation in breast cancer. A and B,** MTT (A) and colony formation (B) analysis of cell proliferation in MCF10A cells transfected with miR-190 inhibitor and MDA-MB-231 cells transfected with miR-190 mimic, as well as in control cells.


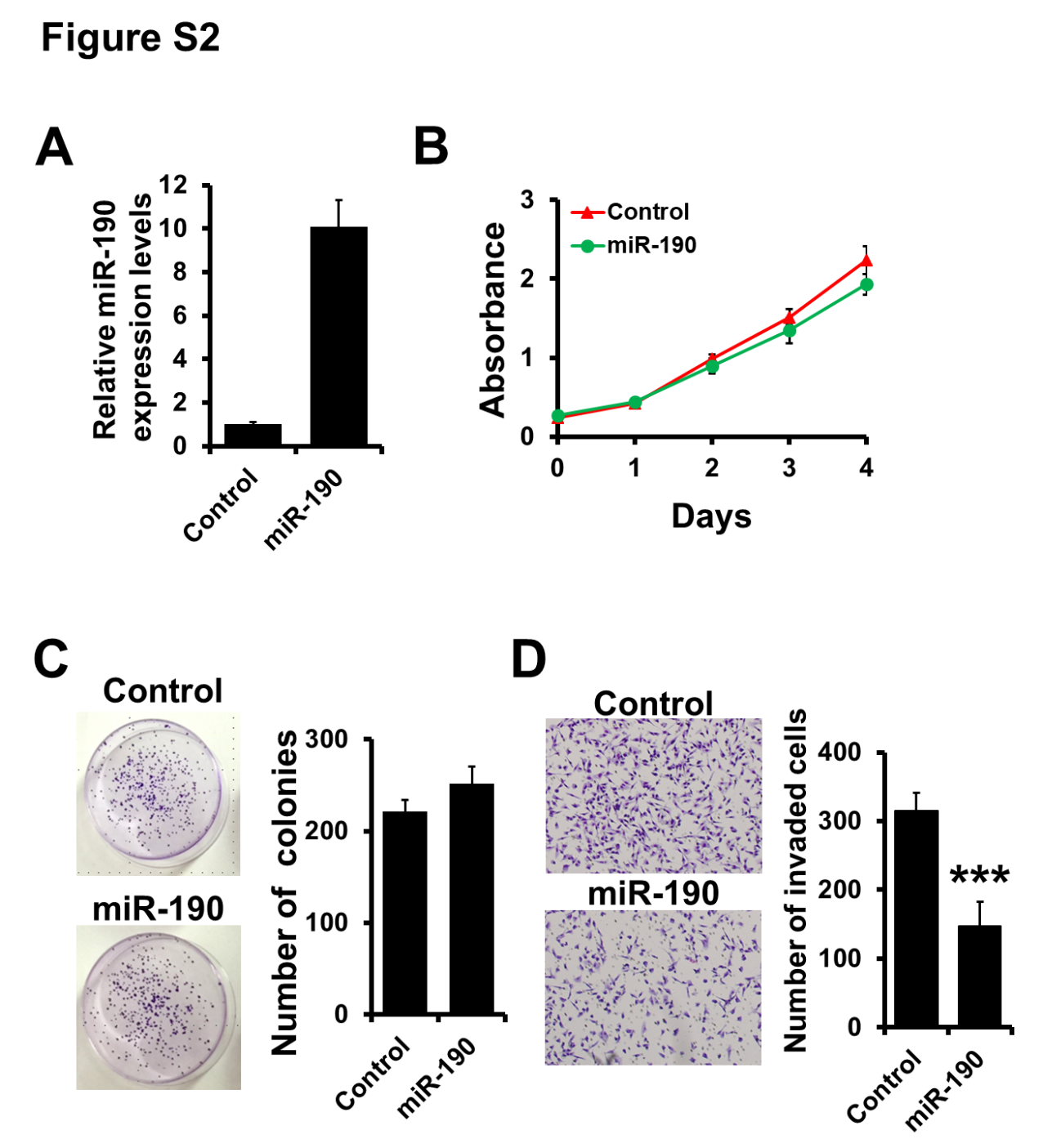


**Figure S2. miR-190 promotes cell invasion but does not affect cell proliferation in MDA-MB-231-luc cells. A,** The expression of miR-190 in MDA-MB-231-luc cells with stable overexpression of miR-190, as determined by RT-qPCR. **B and C,** MTT (B) and colony formation (C) analysis of cell proliferation in cells as in (A). **D,** Transwell invasion assay of cells as in (A).


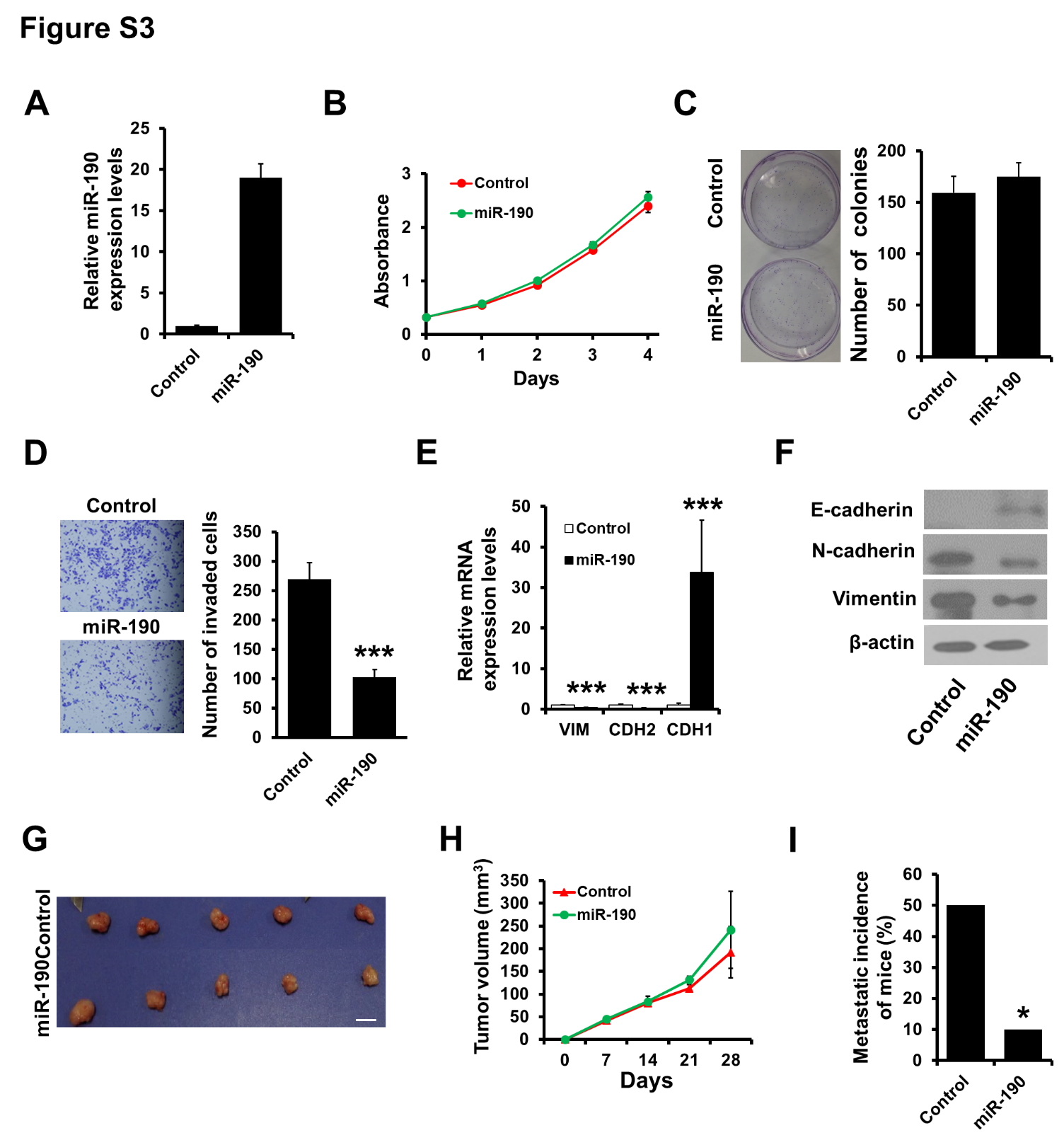


**Figure S3. miR-190 suppresses breast cancer metastasis and EMT phenotype in BT549 cells. A,** The expression of miR-190 in BT549 cells with stable overexpression of miR-190, as determined by RT-qPCR. **B and C,** MTT (B) and colony formation (C) analysis of cell proliferation in cells as in (A). **D,** Transwell invasion assay of cells as in (A). **E and F,** The mRNA (E) and protein (F) expression of EMT markers in cells as in (B) were detected by RT-qPCR and western blotting. **G,** Representative photos of the tumors formed by BT549-miR-190 or control cells at harvest time. **H,** Tumor volume of xenograft mice injected with BT549-miR-190 or control cells at the indicated times. **I,** Metastatic incidence of xenograft mice injected with BT549-miR-190 or control cells. Scale bar, 1 cm. ****P* < 0.001, **P* < 0.05.

**Table S1. Primers used for RT-qPCR.**

| Genes | Forward (5’- to 3’-) | Reverse (5’- to 3’-) |
| --- | --- | --- |
| ACTB | AGGCCAACCGCGAGAAGATGACC | GAAGTCCAGGGCGACGTAGCAC |
| CDH1 | CAGCCACAGACGCGGACGAT | CTCTCGGTCCAGCCCAGTGGT |
| CDH2 | TCGCCATCCAGACCGACCCA | GCAGTTGACTGAGGCGGGTGC |
| Vimentin | ACGTTCGTCAGCAGTATGAAA | GTTAGCAGCCTCAGAGAGGTC |
| SMAD2 | GTTCCTGCCTTTGCTGAC | TCTCTTTGCCAGGAATGCTT |
